# Multiple-Level Regulation of 2,4-Diacetylphloroglucinol Production by the Sigma Regulator PsrA in *Pseudomonas fluorescens* 2P24

**DOI:** 10.1371/journal.pone.0050149

**Published:** 2012-11-29

**Authors:** Xiaogang Wu, Jiucheng Liu, Wei Zhang, Liqun Zhang

**Affiliations:** 1 Department of Plant Pathology, China Agricultural University, Beijing, People's Republic of China; 2 Key Laboratory of Plant Pathology, Ministry of Agriculture, Beijing, People's Republic of China; Cairo University, Egypt

## Abstract

**Background:**

*Pseudomonas fluorescens* 2P24 is a rhizospheric bacterium that aggressively colonizes the plant roots. It produces the antibiotic 2,4-diacetylphoroglucinol (2,4-DAPG), which contributes to the protection of various crop plants against soil borne diseases caused by bacterial and fungal pathogens. The biosynthesis of 2,4-DAPG is regulated at the transcriptional level in the expression of the *phlACBD* operon as well as at the posttranscriptional level by the Gac/Rsm signal transduction pathway. However, the detailed mechanism of such regulation is not clear.

**Methodology/Principal Findings:**

In this study, we identified a binding site for the sigma regulator PsrA in the promoter region of the *phlA* gene. Electrophoretic mobility shift experiments revealed direct and specific binding of PsrA to the *phlA* promoter region. Consistent with the fact that its binding site locates within the promoter region of *phlA*, PsrA negatively regulates *phlA* expression, and its inactivation led to significant increase in 2,4-DAPG production. Interestingly, PsrA also activates the expression of the sigma factor RpoS, which negatively regulates 2,4-DAPG production by inducing the expression of the RNA-binding protein RsmA.

**Conclusions/Significance:**

These results suggest that PsrA is an important regulator that modulates 2,4-DAPG biosynthesis at both transcriptional and posttranscriptional levels.

## Introduction

Rhizosphere-inhabiting fluorescent *Pseudomonas* spp. is a group of ubiquitous root colonizing bacteria with remarkable propensity of interacting with plant roots and protecting the roots against pathogenic microorganisms [1]–[5]. The ability of pseudomonads to suppress soil borne pathogens mainly depends on their ability to secrete secondary antibiotic metabolites, such as pyrrolnitrin, phenazines, pyoluteorin, hydrogen cyanide, and 2,4-diacetylphloroglucinol (2,4-DAPG) [1], [4], [6], [7]. Among these antimicrobial compounds, 2,4-DAPG is a phenolic derivative with antifungal, antibacterial, antiviral, and phytotoxic properties that has been intensively studied [1], [8]–[11]. Besides its anti-microbial activity, 2,4-DAPG induces systemic resistance in plants and promotes exudation of amino acids from roots [12], [13].

The products of the four-gene operon *phlACBD* are responsible for the biosynthesis of 2,4-DAPG. Among these proteins, PhlD shows structural similarity with type III polyketide synthase, which is critical for the biosynthesis of monoacetylphloroglucinol (MAPG). PhlA, PhlC and PhlB are required for the transacetylation of MAPG to produce DAPG [6], [14], [15]. Biosynthesis of 2,4-DAPG is regulated by multiple factors. First, expression of the *phlACBD* operon is controlled by the divergent *phlF* gene, which codes for a transcriptional repressor [16]. Repression by PhlF is achieved by its interaction with the specific binding site, *pho*, located in the promoter region of *phlA* [17]. Second, maximal production of 2,4-DAPG occurs in the late exponential phase or stationary phase and is regulated by a number of additional factors, including the GacS/GacA two-component system [4], [7], [18], the small RNA-binding proteins RsmA and RsmE [19], [20], the sigma factors RpoD, RpoN and RpoS [21]–[23], and the resistance-nodulation-division efflux pump EmhABC [24].

Differing from many bacterial pathogens of plants or animals in which the highly conserved GacS/GacA two-component system regulates important virulence traits [25], [26], this system controls the disease suppression ability in plant-beneficial pseudomonads [18], [25], [27]. GacS is a sensor histidine kinase, which by responding to yet unknown signals, undergoes autophosphorylation. The signals are then relayed to the response regulator GacA by phosphotransfer, leading to the activation of a number of diverse genes, including non-coding small RNAs. For examples, expression of *csrB* and *csrC* in *Escherichia coli*, and *rsmZ*, *rsmY* and *rsmX* in *P. fluorescens* are regulated in this manner [25], [28]–[31]. These small RNAs have a high binding affinity for small RNA-binding proteins of the CsrA/RsmA family, which negatively controls the expression of extracellular enzymes [20], [25], [28], [32], [33].

In *E. coli*, RpoS influences the expression of many genes during the transition from exponential to stationary phase to produce proteins usually associated with resistance to starvation or osmotic stress [34]. In *Pseudomonas* spp., mutations in *rpoS* lead to pleiotropic phenotypes, such as reduction in bacterial survival under environmental stress or alterations in the production of antibiotics pyoluteorin and 2,4-DAPG [21], [35], [36]. However, the role of *rpoS* in the production of secondary metabolites is complex and displays great variations among bacterial species and the products involved. For example, in *P. fluorescens* strain Pf-5, mutations in *rpoS* lead to a decrease of pyrrolnitrin production, which is accompanied by an increase in the production of pyoluteorin and 2,4-DAPG [21].

The level of RpoS in bacterial cells increases considerably when the culture enters the stationary phase [35]; the GacS/GacA two-component system is necessary for the timely expression and accumulation of RpoS during the transition from exponential growth to the stationary phase, indicating that RpoS is a component of the regulatory circuits involved in GacS/GacA [37], [38].

In this study, we describe the identification and characterization of PsrA, a new regulator involved in 2,4-DAPG synthesis in *P. fluorescens* 2P24. We show that PsrA negatively controls the *phlA* gene at the transcriptional level via direct binding to an operator in the *phlA* promoter region as well as at the posttranscriptional level by influencing the expression of RpoS and RsmA.

## Materials and Methods

### Bacterial strains, plasmids, and growth conditions

The bacterial strains and plasmids used in this study are listed in Table S1. *E. coli* was grown in LB medium at 37°C, *P. fluorescens* was cultured in LB medium or ABM minimal medium [39] at 30°C. When necessary, antibiotics were added at the following concentrations: ampicillin at 50 µg ml^−1^, chloramphenicol at 20 µg ml^−1^, gentamicin at 30 µg ml^−1^, kanamycin at 50 µg ml^−1^, and tetracycline at 20 µg ml^−1^.

### DNA manipulations

Plasmid and chromosomal DNA isolation, restriction enzyme digestion, ligation, and gel electrophoresis were performed by standard methods [40]. Plasmids were introduced into *E. coli* via chemical transformation, and into *P. fluorescens* strains via biparental mating or electroporation [41]. Nucleotide sequences were determined by Sunbiotechnology Co. Ltd (Beijing, China) and were analyzed using BLAST [42].

### Construction of bacterial mutants

**Figure 1:**
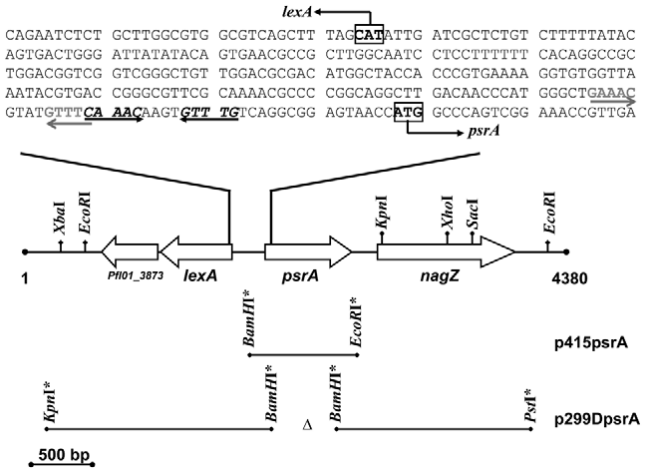
Schematic of the physical location of the *psrA* gene in *Pseudomonas fluorescens* 2P24. *lexA*, gene encoding LexA repressor protein; *psrA*, *Pseudomonas* sigma regulator; *nagZ*, gene encoding β-*N*-acetyl-_D_-glucosaminidase; other gene names refer to the gene symbols as annotated in the *Pseudomonas fluorescens* Pf0-1 genome (GenBank accession no. CP000094). The bars indicate the fragments cloned into the vector pHSG299 to obtain p299DpsrA. The fragment inserted into pRK415 was used to complement the *psrA* mutant. Two putative PsrA binding sites are indicated with inverted arrows. Δ, the region deleted in the mutant PM113 and in plasmid p229DpsrA. Artificial restriction sites are marked with asterisks.

To generate the *psrA* gene mutant PM113, two fragments flanking the *psrA* gene were amplified by PCR using the primers psrA50/psrA1770 and psrA2360/psrA4250, respectively (Table S1). After digestion with relevant restriction enzymes, the two PCR fragments were cloned into pHSG299 (TaKaRa), producing p299DpsrA (Fig. 1). Allelic exchange using p299DpsrA with the wild-type 2P24 resulted in mutant PM113. The mutant was verified by diagnostic PCR. To construct the plasmid for complementation, the coding region of the *psrA* gene was PCR-amplified using primers psrA1660/psrA2410 and cloned into pRK415 [43] to yield p415-psrA (Fig. 1). Plasmid pJN-psrA was constructed by PCR amplification of *psrA* using the PsrA-EcoRI and PsrA-XbaI primer pair and 2P24 genomic DNA as template. The *Eco*RI-*Xba*I restriction fragment was cloned into pJN105 [44], resulting in the *psrA* expression vector pJN-psrA.

To construct a C-terminal vesicular stomatitis virus glycoprotein (VSV-G) epitope-RsmA fusion, a PCR-generated fragment with the sequence 5′-TATACAGATATTGAAATGAATAGATTAGGAAAA-3′ inserted in-frame to the 3′ end of the *rsmA* gene was cloned into pK18mobGII [45]. The resulting plasmid pK18RsmAVSV was verified by sequencing and mobilized into strains 2P24 and the *rpoS* mutant PM303 to generate strains PM114 and PM304, respectively. The plasmid pK18PhlAVSV which contains a -VSV-G fusion was constructed similarly, and was mobilized into strains 2P24, PM113, and PM601 to generate strains PM305, PM306, and PM307, respectively. All mutations were verified by DNA sequencing.

### Construction of reporter fusions and β-galactosidase assays

To construct *psrA*-*lacZ*, *rpoS*-*lacZ*, *rsmA*-*lacZ*, *rsmE*-*lacZ*, *rsmY*-*lacZ* and *rsmX*-*lacZ* transcriptional fusions, the DNA fragments containing the upstream region of each of these genes (300-bp for *psrA*, 470-bp for *rpoS*, 530-bp for *rsmA*, 280-bp for *rsmE*, 330-bp for *rsmY*, and 270-bp for *rsmX*) were PCR amplified using the appropriate primer sets (Table S1). Each of these *cis*-acting regions was inserted as a *Bam*HI or a *Bgl*II fragment into pRG970Gm [46] to place them upstream of a promoterless *lacZ* gene. Plasmid p970Gm-rsmZMp was constructed by PCR amplification using primer pair rsmZMp and RsmZ-P3361 and inserted into pRG970Gm as a *Bam*HI fragment. In this construct, an upstream activating sequence (UAS) in the *rsmZ* promoter was deleted. These plasmids were introduced into appropriate *P. fluorescens* strains by electroporation. β-galactosidase activity of the testing strains was determined by using the Miller method [47].

### Site-directed mutagenesis of the *phlA* and the *rpoS* promoters

To introduce specific mutations into the *phlA* promoter, a 740-bp *Bam*HI fragment containing the *phlA* promoter region was excised from p970Gm-phlAp and inserted into pHSG399 to create p399phlAp. Oligonucleotides containing the designed mutations (primer pairs phlAMGF-phlApM and phlADTF-phlApM) were used to generate p399phlAp derivatives by inverse PCR (QuickChange site-directed mutagenesis kit; Stratagene). The mutated nucleotides were confirmed by DNA sequencing. The obtained p399phlAp derivatives were digested with *Bam*HI and the fragments containing the mutated *phlA* promoter were then inserted into pRG970Gm to generate p970Gm-phlApD3T and p970Gm-phlApM3G, respectively.

A 800-bp fragment containing the promoter of *rpoS* amplified by PCR with primer pairs rpoSp1 and rpoSp2, was inserted into pHSG399 to obtain the plasmid p399rpoSp. Oligonucleotides containing the designed mutation (primer pair RpoSMF and RpoSMR) were used to generate p399rpoSp derivative by inverse PCR (see above). The mutated nucleotides were confirmed by DNA sequencing.

### Expression and purification of the PsrA protein

The predicted ORF of *psrA* was amplified by PCR with primers psrA-NdeI and psrA-XhoI (Table S1). After *Nde*I-*Xho*I digestion, the PCR product was cloned into pET-22b(+) to give pET-PsrA, which was introduced into *E. coli* BL21(DE3) (Novagen) for the production of His_6_-PsrA. To induce protein expression, IPTG was added to *E. coli* cultures grown to an optical density at 600 nm (OD_600_) of 0.6 at a final concentration of 0.8 mM. His_6_-PsrA was purified by using a nickel affinity column (Amersham Biosciences) according to the manufacturer's instructions. Recombinant proteins were eluted by 200 mM imidazole, and the purity was determined by sodium dodecyl sulfate-polyacrylamide gel electrophoresis (SDS-PAGE) followed by Coomassie brilliant blue staining. Protein concentration was determined by Nanodrop ND-1000 (Thermo Scientific) absorbance at 280 nm and by the Bicinchoninic acid (BCA) assay.

### Electrophoretic mobility shift assay

The upstream DNA fragments of the *psrA*, *phlA*, or *rpoS* genes containing the putative PsrA-binding sequences were amplified by PCR using the appropriate primer sets, respectively (Table S1). The DNA fragments were purified using a QIAquick gel extraction kit (Qiagen). Protein-DNA interaction assays were performed in 20 µl of 1× binding buffer (20 mM HEPES, pH 7.6; 1 mM EDTA; 10 mM (NH4)_2_SO_4_; 1 mM DTT; 150 mM KCl, 5% [wt/vol] glycerol). The reaction mixtures were incubated at room temperature for 20 min. Each binding reaction was loaded onto an 8% native polyacrylamide gel and ran for 2 h at 90 Volts. Gels were stained with SYBR Green as recommended by the manufacturer of the EMSA kit E33075 (Invitrogen).

### Production of PsrA antibodies

Polyclonal antibodies against PsrA of *P. fluorescens* 2P24 were produced in a mouse by subcutaneous immunization with 200 µg of the purified recombinant His_6_-PsrA, and this initial immunization was followed by additional immunizations at 3 and 4 weeks, respectively. Six days after the last injection, the blood of the immunized mouse was collected, and its serum was used for Western blot analysis.

### Western blot analysis

Cell lysates of the *P. fluorescens* 2P24 or its derivatives were prepared by sonication in TNT buffer (10 mM Tris-HCl [pH 8.0], 150 mM NaCl, 0.05% Tween-20) (Sambrook and Russell, 2001), the soluble fractions separated by SDS-PAGE were transferred onto polyvinylidene fluoride (PVDF) membrane (Millipore). After blocking with 5% milk in PBST (PBS containing 0.02% Tween-20), membranes were incubated with the appropriate primary antibody: anti-PsrA (1∶1,000), anti-VSV-G antibody (1∶2,000; Sigma) and anti-3-phosphoglycerate kinase (PGK) (1∶2,000; Invitrogen). Blots were washed with PBST, probed with an anti-mouse horseradish peroxidase conjugated secondary antibodies (1∶5,000; Sigma). The resulting blots were incubated for 1 min in ECL reagent and detected using O-MAT X-ray film (Kodak).

### Extraction and quantification of the 2,4-DAPG production

*P. fluorescens* 2P24 and its derivatives were cultivated with shaking in 30 ml LB liquid media at 140 rpm at 30°C. 2,4-DAPG was extracted from the culture supernatant and was quantified by HPLC as described previously [10].

### Nucleotide sequence accession number

The sequence of the *psrA* gene from strain 2P24 has been deposited in the GenBank database under accession no. HQ392504.

## Results

### The *phlA*-*phlF* intergenic region in *P. fluorescens* 2P24 contains a PsrA binding site

The importance of inverted repeat sequences for regulatory protein binding has been established for numerous regulators [48], [49]. Previous studies have revealed the presence of several palindromic sequences in the *phlA*-*phlF* intergenic region in 2,4-DAPG-producing *P. fluorescens* [17]. A sequence alignment of the *phlA* gene promoter region from strain 2P24 with other well-studied 2,4-DAPG producing *Pseudomonas* strains revealed a very well conserved palindromic sequence GAAACN_5_GTTTC (Fig. S1). This element is a potential recognition sequence for PsrA (*Pseudomonas* sigma regulator), which was previously identified as a positive regulator of *rpoS* expression in *P. putida* WCS358 and *P. aeruginosa* PAO1 [50], [51] and was involved in the regulation of type III secretion system and quorum sensing [52], [53]. Our ongoing genome sequence of strain 2P24 revealed that this strain codes for a 237 amino acid PsrA homolog (Fig. 1). This protein is predicted to have a pI of 9.75 and a molecular mass of 26.2 kDa. It contains a helix-turn-helix motif (residues 10 to 56) in its N-terminal portion, which is conserved in members of the TetR family regulatory proteins. PsrA of strain 2P24 is highly similar to predicted PsrA proteins from other pseudomonads, including *P. fluorescens* Pf0-1 (accession number ABA75608; 96% identity), *P. fluorescens* Pf-5 (accession number AAY91237; 93% identity), *P. chlororaphis* PCL1391 (accession number AAM52309, 95% identity), *P. putida* F1 (accession number ABQ79722; 94% identity), *P. syringae* pv. *tomato* DC3000 (accession number AAO56983; 91% identity), and *P. aeruginosa* PAO1 (accession number AAG06394, 87% identity).

**Figure 2:**
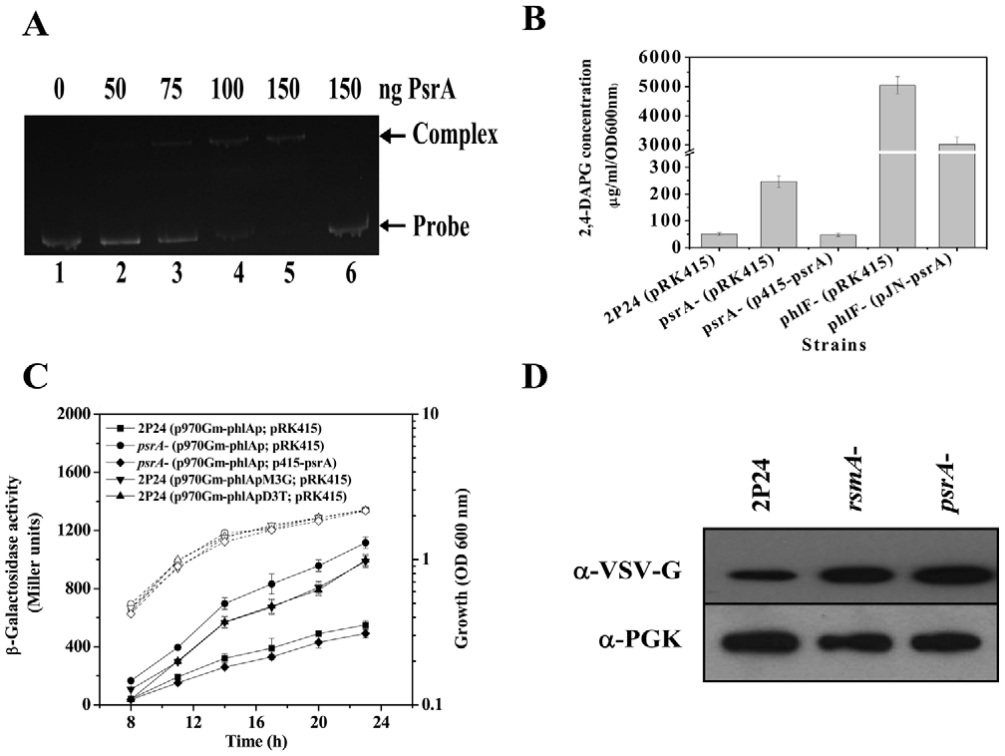
EMSA of PsrA with the *phlA* (30 ng) promoter fragment that contains PsrA-binding sequence showing formation of a PsrA-DNA complex. Lane 1, DNA probe alone; lanes 2–5, DNA probe incubated with 50, 75, 100, or 150 ng PsrA, respectively; lane 6, the mutagenized DNA probe from p399phlAp derivative (a 3-bp substitution [GGG for TTT] in the *phlA* promoter) incubated with 150 ng PsrA (A). Biosynthesis of 2,4-DAPG in strain 2P24 and its *psrA* and *phlF* mutants was assayed by HPLC (B). For transcriptional assay, strain 2P24 and its *psrA* mutant carrying p970Gm-phlAp (wild type *phlA*-*lacZ*), p970Gm-phlApM3G (PsrA box mutTTT *phlA*-*lacZ*) or p970Gm-phlAD3T (PsrA box ΔTTT *phlA*-*lacZ*) were grown in LB, and β-galactosidase activities were determined (C). Analysis of PhlA-V levels in strain 2P24 and the *psrA* mutant by immunoblotting. An antibody directed against 3-phosphoglycerate kinase α (α-PGK) is used as a loading control in this and later blots (D). All experiments were performed in triplicate, and the mean values ±SD are indicated. Growth is indicated by the dotted line.

The presence of a putative PsrA-binding sequence in the *phlA* promoter region suggests potential interactions between PsrA and the *phlA* promoter sequence. We tested this hypothesis by electrophoretic mobility shift assay (EMSA) using the purified His_6_-PsrA protein and DNA fragments of the promoter. A mobility shift was observed when His_6_-PsrA was incubated with the *phlA* promoter (Fig. 2A). Importantly, no shift was detected when mutations disrupting the putative binding site were introduced (Fig. 2A). These results indicate that PsrA may regulate the expression of the *phlA* gene by directly interacting with *phlA* promoter.

### PsrA negatively regulates 2,4-DAPG production at transcriptional level

In LB medium, the *psrA* mutant PM113 produced higher levels of an uncharacterized red pigment, which is a characteristic phenotype associated with the production of the antibiotic 2,4-DAPG (data not shown) [3], [17]. Further quantification by HPLC showed that the 2,4-DAPG levels in culture supernatant of the *psrA* mutant were about 5-fold higher than that of the wild type strain. Such increase could be repressed by introducing the *psrA* gene into the mutant (Fig. 2B). An earlier study has shown that inactivation of the *phlF* gene results in overproduction of 2,4-DAPG in *P. fluorescens* 2P24 [54]. Interestingly, overexpression of PsrA in the *phlF* mutant caused dramatic reduction in 2,4-DAPG production (Fig. 2B). To test the influence of PsrA on the *phlA* transcription, p970Gm-phlAp [46] carrying a *phlA-lacZ* transcriptional fusion was transformed into the *psrA* mutant and its parental strain 2P24. When the cultures were in the stationary phase, expression of *phlA* increased about 3-fold in the *psrA* mutant compared to that of strain 2P24 (Fig. 2C). Disruption of the PsrA box led to expression of the fusion irresponsive to the regulatory protein (Fig. 2B). The expression of the *phlF-lacZ* transcriptional fusion was measured in a *psrA*-deficient mutant and no difference was detected between the mutant and 2P24 (data not shown). In addition, Western blot assay using a chromosomal *vsv-phlA* fusion showed that levels of PhlA protein increased in the *psrA* mutant compared to the wild type strain (Fig. 2D). Together, these results indicate that PsrA controls 2,4-DAPG production by directly regulating the transcription of the 2,4-DAPG biosynthetic operon.

### PhlF and PsrA expressed differently in *P. fluorescens* 2P24

**Figure 3:**
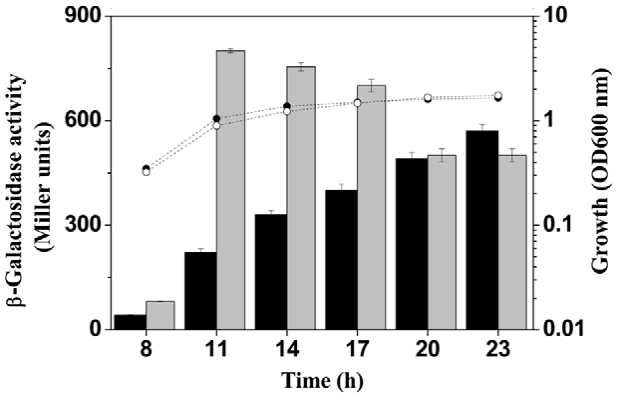
The transcriptional fusions *psrA*-*lacZ* and *phlF*-*lacZ* were introduced into *P. fluorescens* 2P24, respectively. Bacteria were grown in LB medium, and absorbance was measured at 600 nm (solid circles, *psrA*-*lacZ*; open circles, *phlF*-*lacZ*). Expression of the fusions was assessed by measuring levels of β-galactosidase. Black shading represents *psrA*-*lacZ* expression, and grey shading represents *phlF*-*lacZ* expression. Triplicate cultures were assayed and the standard deviations are presented with error bars.

PhlF is a specific transcriptional repressor that regulates the expression of the 2,4-DAPG biosynthetic operon by binding to the *pho* site located about 120-bp downstream of the PsrA recognization site in the *phlA* gene promoter region (Fig. S1). To compare the expression of *psrA* and *phlF*, *lacZ* fusion of these two genes was introduced into strain 2P24. The *psrA* gene expressed in a cell density-dependent manner and reached its maximum in the stationary phase, whereas the transcription of the *phlF* gene reached the highest level earlier in logarithmic phase (Fig. 3). Differential expression of PhlF and PsrA during cell growth implied their distinctive effects on *phlA* transcription. PhlF is likely a major repressor during the early growth phase. In agreement with this notion, 2,4-DAPG normally begins to accumulate during the transition from logarithmic to stationary phase [17].

PsrA regulates the expression of itself in *P. putida*, *P. aeruginosa*, and *P. syringae* by directly binding to its own promoter [51]. In strain 2P24, two putative PsrA-binding sequences ( GAAACGTATGTTTC and CAAACAAGTGTTTG ) are present in the upstream region of the *psrA* gene (Fig. 1). By EMSA, we found that PsrA directly binds its promoter region (Fig. S2A). Consistently, the *psrA-lacZ* fusion expressed at a higher level in the *psrA* defective mutant (Fig. S2B).

### PsrA positively regulates the translational regulator RsmA

**Figure 4:**
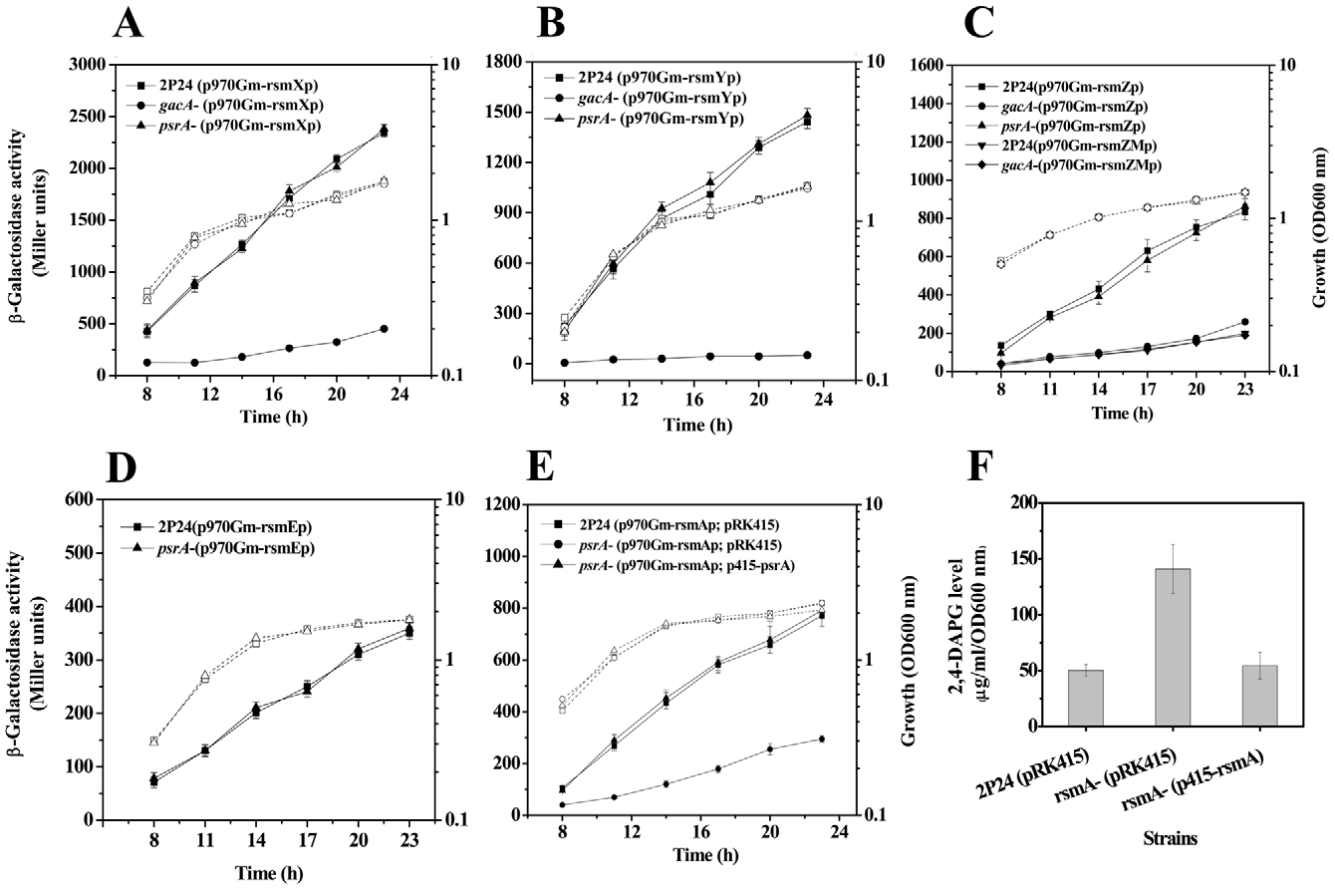
Transcription of small non-coding RNA genes *rsmX* (A), *rsmY* (B), and *rsmZ* (C) and their cognate regulator genes *rsmE* (D) and *rsmA* (E) in *P. fluorescen*s 2P24, its *psrA* mutant and its *gacA* mutants. (F) HPLC analysis of 2,4-DAPG production by strain 2P24 and its *rsmA* mutant. All experiments were performed in triplicate, and the mean values ±SD are indicated. Growth is indicated by the dotted line.

In some *Pseudomonas* strains, biosynthesis of 2,4-DAPG is tightly regulated at both transcriptional and post-transcriptional level [25], [27]. Multiple factors of the Gac/Rsm signal transduction pathway, including the GacS/GacA system, the small RNAs RsmX, RsmY, and RsmZ, and the translational repressors RsmA and RsmE are important components of regulatory circuit that controls the production of 2,4-DAPG at posttranscriptional level [25], [33]. In *P. fluorescens* 2P24, three small RNAs, RsmX, RsmY, and RsmZ, and two small RNA-binding proteins RsmA and RsmE are present in the draft genome sequence. To determine whether PsrA influences expression of these genes, we measured the transcription of these genes in the *psrA* mutant. Although no difference for *rsmZ*, *rsmY, rsmX* or *rsmE* was observed (Fig. 4A–D), the expression of *rsmA* was significantly lower in the mutant (Fig. 4E). Consistent with this observation, the *rsmA* mutant produced significantly higher amounts of PhlA protein (Fig. 2D) and 2,4-DAPG (Fig. 4F) than the wild type strain 2P24. This increase of 2,4-DAPG can be brought to the wild type level by expressing *rsmA* in the mutant (Fig. 4F), further validating the negative role of RsmA in 2,4-DAPG production. Thus, PsrA positively regulates the transcription of the *rsmA* gene, which in turn negatively controls the biosynthesis of 2,4-DAPG.

### The effect of PsrA on *rsmA* gene expression is mediated by the sigma factor RpoS

**Figure 5:**
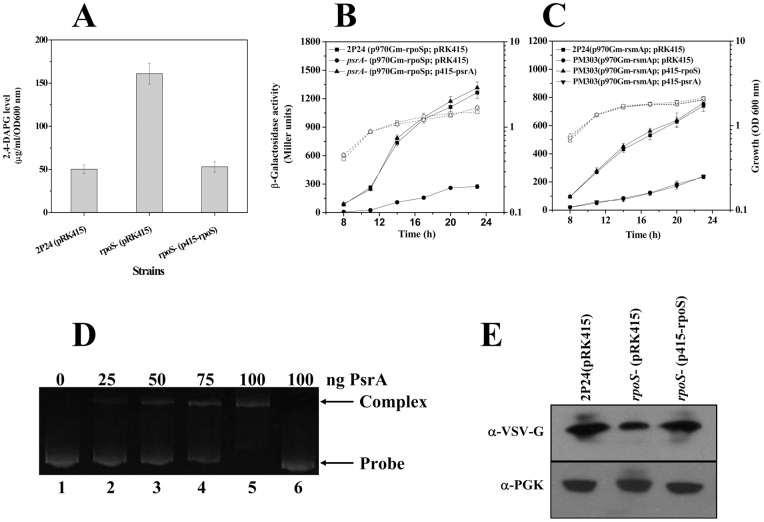
RpoS regulates the 2,4-DAPG production via RsmA in *P. fluorescens* 2P24. Biosynthesis of 2,4-DAPG in strains 2P24 and its *rpoS* mutant was assayed by HPLC (A). The expression of the *rpoS* gene is activated by PsrA in strain 2P24 (B). Expression of the *rsmA* gene in the wild type strain 2P24 and the *rpoS* mutant PM303 (C). Binding assay of PsrA to the *rpoS* promoter. 30 ng DNA probe was incubated with increasing amounts of PsrA. Lane 1, DNA probe alone; lanes 2–5, DNA probe incubated with 25, 50, 75, or 100 ng PsrA, respectively; lane 6, the mutated DNA probe from p399rpoSp derivative (a 3-bp substitution [GGG for TTT] in the *rpoS* promoter) incubated with 100 ng PsrA (D). Western blot analysis of RsmA-V in strain 2P24 and the *rpoS* mutant (E).

The absence of a PsrA binding site in the promoter region of *rsmA* suggests an indirect effect of PsrA on *rsmA* transcription (data not shown). A previous study has shown that RpoS positively regulates *rsmA* gene in *Pectobacterium carotovarum* and its homolog CsrA in *E. coli* [55]. Further, RpoS is a negative regulator for 2,4-DAPG production in *P. fluorescens* CHA0 and Pf-5 [19], [21]. In *P. fluorescens* 2P24, the production of 2,4-DAPG in the *rpoS* mutant increased about 3-fold when compared with the wild type strain (Fig. 5A). We therefore examined whether RpoS regulates *phlA* expression. The *phlA-lacZ* transcriptional fusion in the *rpoS* mutant expressed at levels similar to those in the mutant (Fig. S4), suggesting that the negative effects of RpoS on 2,4-DAPG production occurs at post-transcriptional level.

The above observations suggest that in strain 2P24, PsrA regulates *rpoS* expression, which in turn controls *rsmA* expression. Careful inspection of the promoter region of *rpoS* of strain 2P24 revealed the presence of a putative PsrA binding site between −427 and −416 relative to its transcription start site. Incubation of His_6_-PsrA with a DNA fragment containing this region led to the formation of DNA-protein complex, and no shift was detected when mutations disrupting the putative binding site were introduced (Fig. 5D). In the *psrA* mutant, expression of the *rpoS-lacZ* decreased about 4-fold (Fig. 5B), suggesting a positive role of PsrA in *rpoS* transcription. In the *rpoS* mutant, the *rsmA*-*lacZ* transcriptional fusion expressed at a significantly lower level than in the wild type (Fig. 5C). The defect was caused by the loss of RpoS because introduction of a plasmid-borne allele of this gene completely restored the expression of *rsmA* (Fig. 5C). To examine the protein level of RsmA, we created a chromosomal *vsv-rsmA* in the Δ*rpoS* strain background and found that similar to the results from the *lacZ* fusion, VSV-RsmA was produced at a lower level in the mutant (Fig. 5E). The effect of RpoS on *rsmA* was specific because the expression of *rsmE* or the production of RsmE was not affected by *rpoS* deletion (data not shown). Taken together, these results establish that in addition to directly affecting *phlA* transcription, PsrA influences the production of 2,4-DAPG posttranscriptionaly via RpoS and RsmA.

### The effect of PsrA on 2,4-DAPG production is independent of the GacS/GacA system

**Figure 6:**
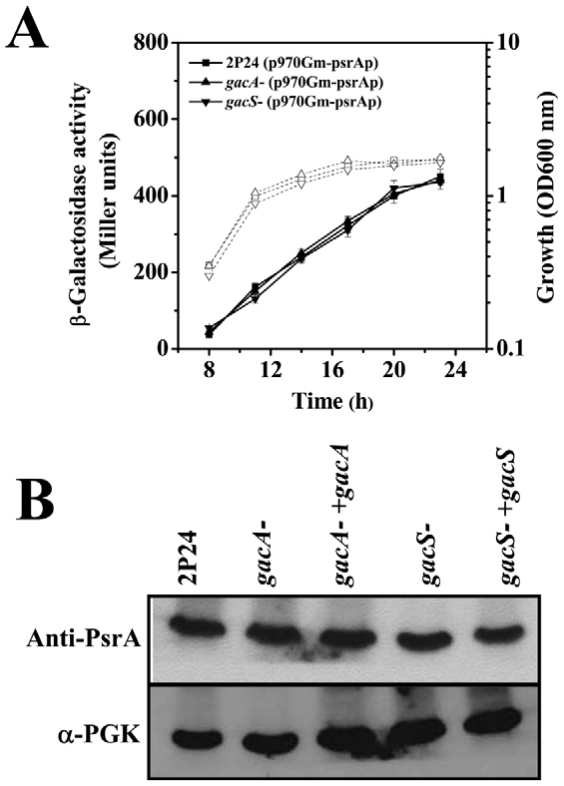
PsrA is not regulated by the GacS/GacA two-component system in *P. fluorescens* 2P24. Transcriptional fusion assay (A) and Western blot analysis (B) demonstrating that the expression of PsrA is not altered in the *gacA* mutant or in the *gacS* mutant.

In *Pseudomonas* spp., components of the Gac/Rsm signal transduction pathway includes the GacS/GacA two-component system, the noncoding small RNAs and the translational repressors. Among these, the GacS/GacA system functions as a global regulatory system which positively regulates transcription of the noncoding small RNAs by binding to a conserved UAS in the promoter region [29], [31]. In *P. chlororaphis*, the transcription of *psrA* was abolished in the *gacS* mutant, suggesting that the *psrA* gene in this bacterium was under the regulation of the two-component system [56]. To determine whether *psrA* was regulated by the GacS/GacA system, we measured the expression of the *psrA-lacZ* fusion in a GacS/GacA-deficient mutant and no difference was detected between the mutant and the wild type strain. Similar results were obtained when the protein levels of PsrA were probed (Fig. 6). Thus, the GacS/GacA two-component system has no effect on the expression of the *psrA* gene in this bacterium.

## Discussion

The antibiotic 2,4-DAPG is one of the major weapons to inhibit the growth of pathogenic bacteria and fungi by some biocontrol *P. fluorescens* strains. *P. fluorescens* mutants unable to produce 2,4-DAPG are defective in its protection against black root rot of tobacco, take-all of wheat, and *Pythium* damping-off of sugarbeet [8], whereas strains 2P24 and CHA0 overproducing 2,4-DAPG exhibit increased plant disease suppression ability [57], [58]. However, overproduction of 2,4-DAPG in some *Pseudomonas* strains led to a notable phytotoxicity [54]. Clearly, precise regulation of 2,4-DAPG production is necessary for proper responses to the ever changing soil environment [4]. A series of transcriptional and translational regulators that control 2,4-DAPG production have been identified in various strains of *P. fluorescens*, including CHA0, F113, Pf-5, Q2–87, Q8r1-96, and 2P24. These regulatory factors inclue the specific repressor PhlF [17], the sigma factors RpoS, RpoD and RpoN [15], [21], [23], the H-NS family regulators MvaT and MvaV [59], the oxidoreductase DsbA [60], the RNA binding protein Hfq [46], the resistance-nodulation-division efflux pump EmhABC [24], and the Gac/Rsm signal transduction pathway [25], [27]. However, the relationships among these factors and how they co-ordinate to control antibiotics production are not fully understood.

In *P. fluorescens* 2P24, both PhlF and PsrA are transcriptional repressors for expression of genes involved in 2,4-DAPG production by directly interacting with specific binding box localized on the *phlA* promoter region (Fig. S1). This observation raises the question of why two seemingly redundant repressors are needed for production of this antibiotic. We show here that the expression pattern of *phlF* and *psrA* is clearly distinct (Fig. 3), and the 2,4-DAPG production in *phlF*- and *psrA*-negative mutants is very different (Fig. 2B), implying that their roles in the production of 2,4-DAPG may differ greatly. 2,4-DAPG production in the *phlF* mutant is more than 20-fold higher than that in the *psrA* mutant (Fig. 2B), indicating that the pathway-specific regulator PhlF is the dominant regulatory factor for 2,4-DAPG production. Transcriptional analysis showed that the *phlF* expressed at a relatively higher level in the early stages of growth (Fig. 3), which is consistent with its role in preventing 2,4-DAPG production in the early log phase and the fact that the antibiotics usually accumulates in the stationary phase (1, 11). Constitutive expression of the *phlACBD* locus could be a metabolic burden, which can lead to reduction in bacterial growth and its ability to compete with adjacent microorganisms. Therefore, additional repressors are necessary to prevent excessive biosynthesis of 2,4-DAPG after the repression by PhlF is lifted. PsrA might play such a role to restrain the overproduction of 2,4-DAPG, particularly in the stationary phase when the expression of the *psrA* gene was significantly higher (Fig. 3). Consistente with this notion, overexpression of PsrA in *phlF* mutant caused a decrease in 2,4-DAPG production (Fig. 2B). Such a regulatory circuit may allow the bacterium to mount a more efficient and precise regulatory response for balanced production of antibiotics to better adapt to the ever-changing environment. In addition, the presence of the recognition sites for PhlF and PsrA in the *phlA* promoter region of strain 2P24 as well other 2,4-DAPG producing pseudomonads, including *P. fluorescens* F113, Q2–87, CHA0, and Pf-5 (GenBank accession numbers AF497760, U41818, AF207529, and CP000076, respectively) further suggests the importance and conservation of PsrA in the regulation of 2,4-DAPG production (Fig. S1).

Consistent with earlier studies, our results revealed that unlike PhlF which is a specific repressor for the *phlACBD* operon, PsrA is a global regulator that positively regulates the expression of the sigma factor RpoS in bacteria [51], [61]. In other *Pseudomonas* spp., PsrA is known to be involved in the regulation of the type III secretion system (TTSS), quorum sensing, fatty acid degradation, and the production of secondary metabolites [52], [53], [56], [62]. Thus, PsrA participates in diverse regulatory networks involved in various physiological functions. In *P. fluorescens* strain 2P24, in addition to the biosynthesis of 2,4-DAPG, several important biological control traits, such as the production of *N*-acyl-homoserine lactone (AHL), biofilm formation and swarming motility were also significantly impaired in the *psrA* mutant (data not shown). PsrA was previously described as a transcriptional regulator involved in positive regulation of RpoS and in negative autoregulation, by direct binding to the conserved motif (G/CAAACN_2–4_GTTTG/C) in the promoter regions [51]. As also confirmed in this study, direct interaction of PsrA with the *phlA* gene promoter in strain 2P24 requires the similar binding motif -5′GAAACGGATCGTTTC3′- (Fig. S1). Genome searching in *P. aeruginosa* revealed that at least 26 genes contain the PsrA binding motif in their promoter regions [61]. But interestingly, this motif seems not always essential for PsrA function. In *P. aeruginosa*, PsrA is required for the full activation of the TTSS regulatory operon *exsCEBA* by binding to its promoter region despite the absence of a recognizable binding site, but some sequences (GAAAC at the position −56 from the transcriptional start site) was resemble a partial binding site [53]. Thus further identification of PsrA-binding genes in strain 2P24 will help us to better understand the global functions of PsrA in response to the environmental changes.

**Figure 7:**
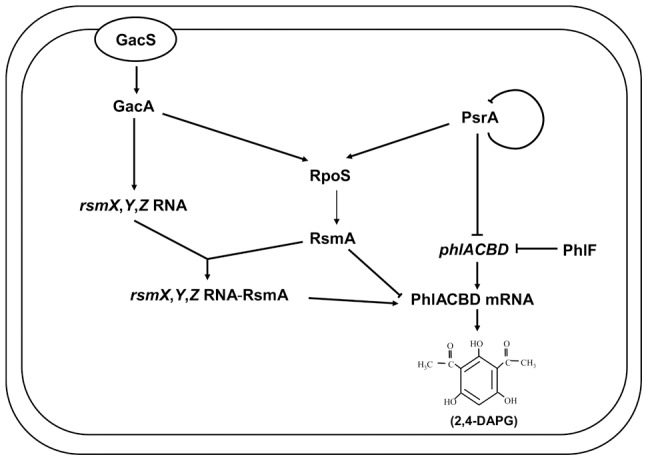
Model for the regulation of 2,4-DAPG biosynthesis in *P. fluorescens* 2P24. In this complex cascade, the sensor GacS is activated by a putative environmental factor. Subsequently, GacS stimulates its cognate regulator GacA. GacA activates small non-coding RNAs, RsmX/Y/Z and sigma factor RpoS, which negatively regulate 2,4-DAPG production at posttranscriptional level through RsmA titration. In addition, PsrA negatively controls transcription of *phlA* and positively regulates *rpoS* by binding to their promoter regions. RpoS has a positive effect on *rsmA* expression. Thus, effect of PsrA on 2,4-DAPG production exerted at both transcriptional and posttranscriptional levels.

Our results indicate that PsrA influences 2,4-DAPG biosynthesis at the post-transcriptional level by activating the expression of the *rsmA* gene through the sigma factor RpoS (Fig. 5). The GacS/GacA two-component system has been known as a major post-transcriptional regulon controlling at least three non-coding small RNAs (RsmZ, RsmY, and RsmX), which together with the translational regulator RsmA/CsrA family proteins, coordinate the expression of secondary metabolites, production of extracellular enzymes, and biocontrol properties [33]. RsmA binds to specific mRNA targets, stabilizing some and inducing the degradation of others [63]. Interestingly, the results in this study differ from a recent finding in *P. fluorescens* CHA0, in which a potential PsrA recognition site (CAAAGN_4_CTTTT) overlapping with the putative GacA binding site in the *rsmZ* promoter was identified and the mutation of this sequence abolished the effect of PsrA on *rsmZ* expression [64]. In *P. fluorescens* 2P24, the GacS/GacA two-component system also positively regulates three small non-coding RNAs (RsmX, RsmY, and RsmZ), and the UAS sequence is necessary for this regulatory role (Fig. 4A–C). However, a PsrA-binding site is not present in the promoter region of *rsmZ* and PsrA did not detectably affect *rsmZ* transcription (Fig. S3; Fig. 4C). These results imply that, although the regulation at the transcriptional level is conserved, the effects of PsrA on 2,4-DAPG biosynthesis at the posttranscriptional level differ distinctively in pseudomonads. Thus, we have proposed a model to illustrate our current understanding of the roles of PsrA in biosynthesis of 2,4-DAPG in *P. fluorescens* 2P24 (Fig. 7). Further study is necessary to identify genes potentially regulated by PsrA to better understand the function of PsrA in other properties of *P. fluorescens*, such as antibiotic production, host interaction, and niche adaptation.

## Supporting Information

Figure S1 Alignment of the *phlA* promoter of *P. fluorescens* 2P24 with homologous sequences in other pseudomonads. The alignment was made using Clustal W (Thompson JD, HigginsD G, Gibson TJ. (1994) CLUSTAL W: improving the sensitivity of progressive multiple sequence alignment through sequence weighting, position-specific gap penalties and weight matrix choice. Nucleic Acids Res 22: 4673–4680). Asterisks denote conserved nucleotides. The putative PsrA binding element and the putative Shine-Dalgarno (SD) site of the *phlA* gene are boxed. RsmA/RsmE proteins are expected to bind the SD region and inhibit the translation of the *phlA* mRNA (Karine L, Elena S, Magnus L, Katja S, Carol SB, et al. (2007) Mechanism of *hcnA* mRNA recognition in the Gac/Rsm signal transduction pathway of *Pseudomonas fluorescens*. Mol Microbiol 66: 341–356). The −10 and −35 promoter element, PhlF binding site: *phO*, and transcription start site of *phlA* have been determined (Abbas A, Morrissey JP, Marquez PC, Sheehan MM, Delany I R, et al. (2002) Characterization of interactions between the transcriptional repressor PhlF and its binding site at the *phlA* promoter in *Pseudomonas fluorescens* F113. J Bacteriol 184: 3008–3016).
(TIF)

Figure S2 EMSA of PsrA with the *psrA* promoter fragment that contains PsrA-binding sequence showing formation of a PsrA-DNA complex. 30 ng DNA probe was incubated with increasing amounts of PsrA (A). β-Galactosidase assay showing the expression profile of a plasmidborne *psrA*-*lacZ* reporter fusion in strain 2P24 and its *psrA* mutant(B). All experiments were performed in triplicate, and the mean values ±SD are indicated. Growth is indicated by the dotted line.
(TIF)

Figure S3 Alignment of the *rsmZ* promoter of *P. fluorescens* 2P24 with homologous sequences in other pseudomonads. The alignment was made using Clustal W as in Fig. S1. Asterisks denote conserved nucleotides. The −10 and −35 promoter element, the putative upstream activating sequence (UAS), the terminator of *rpoS*, the transcription start site and the terminator of *rsmZ*, and the putative PsrA binding site in the *rsmZ* promoter region have been determined (Heeb S, Blumer C, and Haas D. (2002) Regulatory RNA as mediator in GacA/RsmA-dependent global control of exoproduct formation in *Pseudomonas fluorescens* CHA0. J Bacteriol 184:1046–1056; Humair, B., Wackwitz, B., Haas, D. (2010) GacA-Controlled Activation of Promoters for Small RNA Genes in *Pseudomonas fluorescens*. Appl Environ Microbiol 76: 1497–1506).
(TIF)

Figure S4 The expression of *phlA* gene is not regulated by *rpoS* gene at transcriptional level. β-Galactosidase assay showing the expression profile of a plasmidborne *phlA*-*lacZ* reporter fusion in strain 2P24 and its *rpoS* mutant. This experiment was performed in triplicate, and the mean values ±SD are indicated. Growth is indicated by the dotted line.
(TIF)

Table S1 Bacteria strains, plasmids and oligonucleotides used in this study.
(DOC)
