# Supplementary figures and images for: Multiple-Level Regulation of 2,4-Diacetylphloroglucinol Production by the Sigma Regulator PsrA in Pseudomonas fluorescens 2P24
Source: PLoS One. 2012 Nov 29;7(11):e50149. doi: 10.1371/journal.pone.0050149 (PMC3510223; doi:10.1371/journal.pone.0050149)

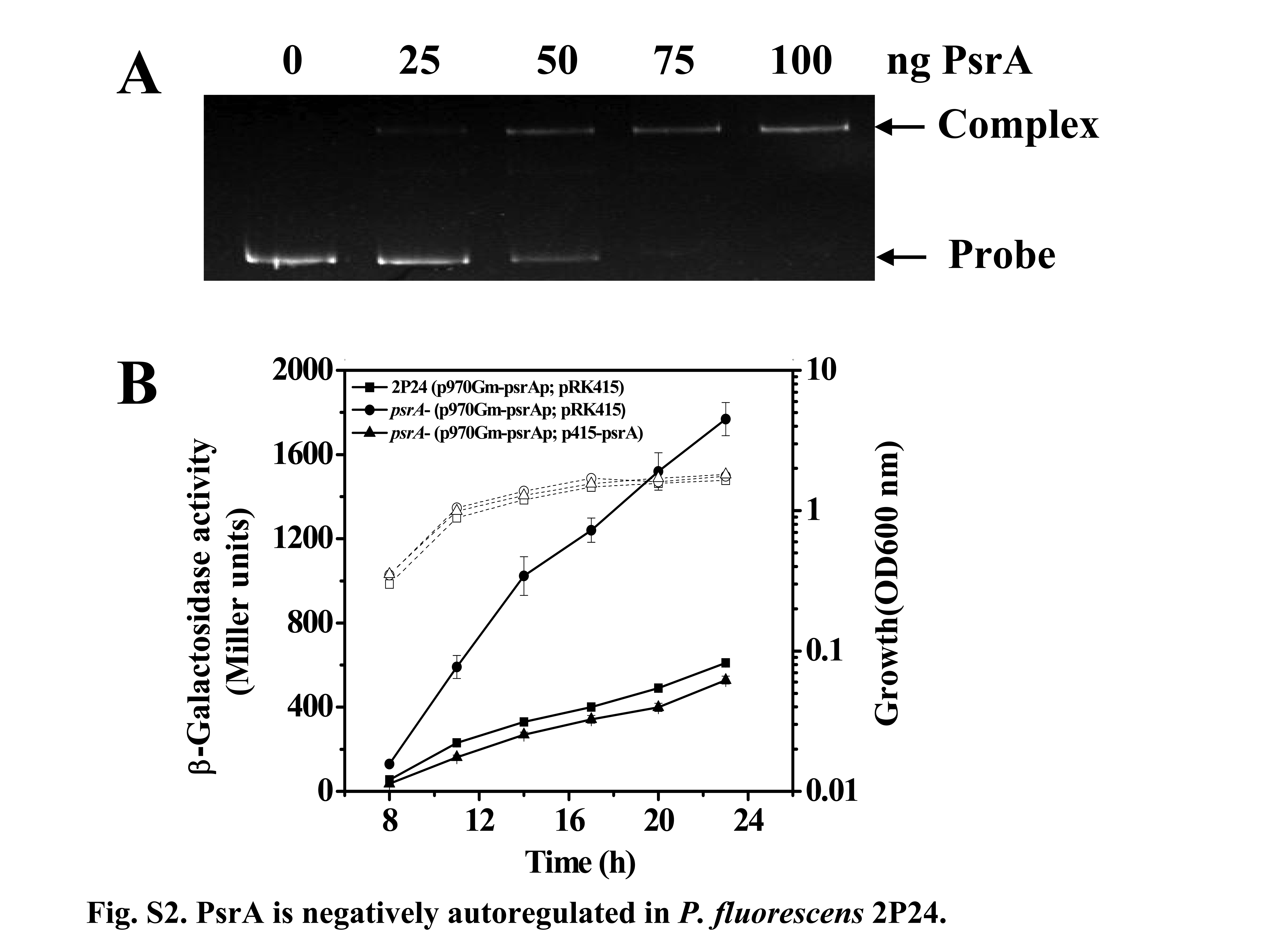

Supplement: Figure S2 — EMSA of PsrA with the psrA promoter fragment that contains PsrA-binding sequence showing formation of a PsrA-DNA complex. 30 ng DNA probe was incubated with increasing amounts of PsrA (A). β-Galactosidase assay showing the expression profile of a plasmidborne psrA-lacZ reporter fusion in strain 2P24 and its psrA mutant(B). All experiments were performed in triplicate, and the mean values ±SD are indicated. Growth is indicated by the dotted line. (TIF) [file pone.0050149.s002.tif]

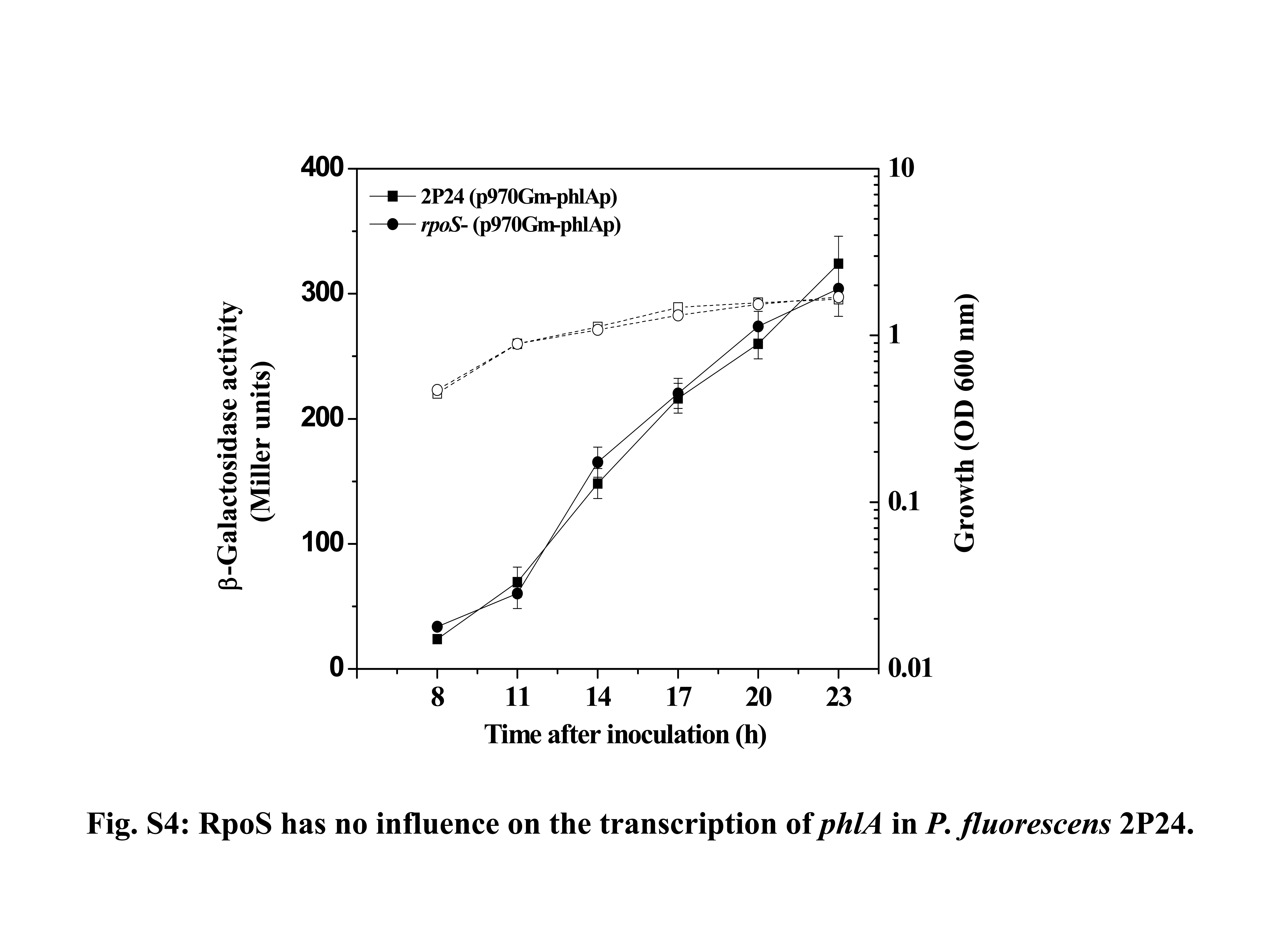

Supplement: Figure S4 — The expression of phlA gene is not regulated by rpoS gene at transcriptional level. β-Galactosidase assay showing the expression profile of a plasmidborne phlA-lacZ reporter fusion in strain 2P24 and its rpoS mutant. This experiment was performed in triplicate, and the mean values ±SD are indicated. Growth is indicated by the dotted line. (TIF) [file pone.0050149.s004.tif]
