# Supplementary material for: Multiple-Level Regulation of 2,4-Diacetylphloroglucinol Production by the Sigma Regulator PsrA in Pseudomonas fluorescens 2P24
Source: PLoS One. 2012 Nov 29;7(11):e50149. doi: 10.1371/journal.pone.0050149 (PMC3510223; doi:10.1371/journal.pone.0050149)
Supplement: Table S1 — Bacteria strains, plasmids and oligonucleotides used in this study. (DOC) [file pone.0050149.s005.doc]

Supplementary Table S1: Bacteria strains, plasmids and oligonucleotides used in this study.

Table S1. Bacteria strains, plasmids and oligonucleotides used in this study

| Strain or plasmid | Description | Reference or source |
| --- | --- | --- |
| *Pseudomonas fluorescens* |  |  |
| 2P24 | Ap^r^; wild type | 41 |
| PM113 | Ap^r^; *psrA* deletion mutant | This study |
| PM114 | Ap^r^; 2P24 with a VSV-G epitope sequence tagged to the C terminus of RsmA | This study |
| PM201 | Ap^r^; *gacA* deletion mutant | Lab stock |
| PM202 | Ap^r^; *gacS* deletion mutant | 41 |
| PM303 | Ap^r^; *rpoS* deletion mutant | Lab stock |
| PM304 | Ap^r^; PM303 with a VSV-G epitope sequence tagged to the C terminus of RsmA | This study |
| PM305 | Ap^r^; 2P24 with a VSV-G epitope sequence tagged to the C terminus of PhlA | This study |
| PM306 | Ap^r^; PM113 with a VSV-G epitope sequence tagged to the C terminus of PhlA | This study |
| PM307 | Ap^r^; PM601 with a VSV-G epitope sequence tagged to the C terminus of PhlA | This study |
| PM601 | Ap^r^; *rsmA* deletion mutant | Lab stock |
| PM911 | Ap^r^; *phlF* deletion mutant | 54 |
| *Escherichia* *coli* |  |  |
| DH5α | *supE44* Δ*lac*U169 (*φ*80*lacZ*ΔM15) *hsdR*17 *recA*1 *endA*1 *gyrA*96 *thi*-1 *relA*1 | 40 |
| S17-1 λpir | λ(pir) *hsdR pro thi*; chromosomally integrated RP4-2 Tc::Mu Km::Tn*7* | Lab stock |
| BL21(DE3) | Expression strain | Novagen |
| Plasmids |  |  |
| pHSG299 | Km^r^; suicide plasmid for *Pseudomonas* spp., used for homologous recombination | TaKaRa |
| p299DpsrA | Km^r^; plasmid pHSG299 carrying a deleted *psrA* gene | This study |
| pHSG399 | Cm^r^; ColE1 origin | TaKaRa |
| p399phlAp | Cm^r^; pHSG399 containing a 750-bp *Bam*HI fragment with the *phlA* promoter | This study |
| p399rpoSp | Cm^r^; pHSG399 containing a 800-bp *Bam*HI fragment with the *rpoS* promoter | This study |
| pK18mobGII | Km^r^; Mob^+^ ColE1 *gusA* | 45 |
| pK18RsmAVSV | Km^r^; pK18mobGII with a VSV-G epitope sequence tagged to the C terminus of RsmA | This study |
| pK18PhlAVSV | Km^r^; pK18mobGII with a VSV-G epitope sequence tagged to the C terminus of PhlA | This study |
| pRK415 | Tc^r^; IncP1 replicon, polylinker of pUC19; Mob^+^ | 43 |
| p415-psrA | Tc^r^; pRK415 containing a 750-bp *Eco*RI-*Bam*HI fragment with the *psrA* gene | This study |
| p415-gacA | Tc^r^; pRK415 containing the *gacA* gene | Lab stock |
| p415-gacS | Tc^r^; pRK415 containing the *gacS* gene | 41 |
| p415-rpoS | Tc^r^; pRK415 containing the *rpoS* gene | Lab stock |
| pJN105 | Gm^r^; Arabinose-inducible gene expression vector; pBRR-1 MCS; *araC*-P_BAD_ | 44 |
| pJN-psrA | Gm^r^; pJN105 containing the *psrA* gene | This study |
| pEG970Gm | Gm^r^; cloning vector containing promoterless *lacZYA* for construction of transcriptional fusion | 46 |
| p970Gm-psrAp | Gm^r^; pRG970Gm containing a *psrA*-*lacZ* transcriptional fusion | This study |
| p970Gm-rsmZp | Gm^r^; pRG970Gm containing an *rsmZ*-*lacZ* transcriptional fusion | Lab stock |
| p970Gm-rsmZMp | Gm^r^; pRG970Gm containing a deletion of the UAS in the *rsmZ* promoter | This study |
| p970Gm-rsmYp | Gm^r^; pRG970Gm containing an *rsmY*-*lacZ* transcriptional fusion | This study |
| p970Gm-rsmXp | Gm^r^; pRG970Gm containing an *rsmX*-*lacZ* transcriptional fusion | This study |
| p970Gm-rsmAp | Gm^r^; pRG970Gm containing an *rsmA*-*lacZ* transcriptional fusion | This study |
| p970Gm-rsmEp | Gm^r^; pRG970Gm containing an *rsmE*-*lacZ* transcriptional fusion | This study |
| p970Gm-rpoSp | Gm^r^; pRG970Gm containing an *rpoS*-*lacZ* transcriptional fusion | This study |
| p970Gm-phlAp | Gm^r^; pRG970Gm containing a *phlA*-*lacZ* transcriptional fusion | 46 |
| p970Gm-phlApD3Tp | Gm^r^; pRG970Gm containing a 3-bp deletion (∆TTT) in the *phlA* promoter | This study |
| p970Gm-phlApM3Gp | Gm^r^; pRG970Gm containing a 3-bp substitution (GGG) in the *phlA* promoter | This study |
| pET-22b(+) | Ap^r^; expression vector | Novagen |
| pET-psrA | Ap^r^; pET-22b(+) carrying the *psrA* gene | This study |
| Oligonucleotides (5′🡢3′) |  |  |
| psrA50 | CGTCTGCAGTTCACTTCCATGTTG |  |
| psrA1770 | ATGGATCCGATGAGGCCATGGCT |  |
| psrA2360 | ATGGATCCGCGAACAACTGCTCG |  |
| psrA4250 | CAGGTACCTGAGCAGGAGTTGAGC |  |
| psrA1660 | CGGAATTCGGCATGGGCGGCTAGCTTAG |  |
| psrA2410 | TAGGATCCACCATGGCCCAGTCGGAAAC |  |
| PsrA-EcoRI | TAGAATTCAGCCTTGGCCAACACCG |  |
| PsrA-XbaI | GATCTAGAAACGAGAGTCGGCATGG |  |
| psrAP1 | TGGGATCCAGAATCTCTGCTTGGCGTG |  |
| psrAP2 | ATGGATCCTGCTCGGCAGCATCGAGAATGC |  |
| rsmEP1 | GAGGATCCCCCGGAAGACATGAGGCATG |  |
| rsmEP2 | CTGGATCCTCGTCACCAATGTTTATGCTTTCA |  |
| rsmYP2830F | ATCCCGGGTTTGGCTACACCGCTTACGC |  |
| rsmYP3160R | ATCCCGGGCAGATCCTACGTGTCCGTG |  |
| rsmZMP | ATGGATCCCATTGCCGGGATAGACGAC |  |
| RsmZ-P3361 | TTGGATCCTTCCCATCGTCCTTGAC |  |
| rpoSP1 | ATGGATCCTTCTAATGGCGTTCTTATTG |  |
| rpoSP2 | CAGGATCCGGCACTTCTTTACTGAGAGC |  |
| RpoSMF | TCTCTTCAAACGGTAGGGGGAATAAAGGA |  |
| RpoSMR | GCCTTCTAATGGCGTTCTTATTGGAAAAT |  |
| RsmX961 | ATTAGATCTAGTCGTGGAGTCGGGTTTCAA |  |
| RsmX1229 | ATTAGATCTGATCCTGAGCACTTCCTGT |  |
| phlA2267 | ACGGATCCAGATCTTAAGGGTTTCTATGGCG |  |
| RsmA2064P1 | ATGGATCCACTGACTTCACCTTCACCGTGCAC |  |
| RsmA2596P2 | CAGGATCCAATAATCAGGCTTTCTGCACACCG |  |
| phlA3010 | GTGGATCCATAAGGATTGGTGCAG |  |
| phlAMGF | GCAATGAAACGGATCGGGGCGTTTTGTA |  |
| phlApM | TTTTCGAGAGAATCCTATACCCTGAGTCTC |  |
| phlADTF | GCAATGAAACGGATCGCGTTTTGTA |  |
| psrA-NdeI | GCGCATATGGCCCAGTCGGAAACCGTTG |  |
| psrA-XhoI | ATACTCGAGAGCCTTGGCCAACACCGGGGCCG |  |
